# Supplementary material for: Transcriptome Analysis Reveals Vimentin-Induced Disruption of Cell–Cell Associations Augments Breast Cancer Cell Migration
Source: Cells. 2022 Dec 13;11(24):4035. doi: 10.3390/cells11244035 (PMC9776984; doi:10.3390/cells11244035)
Supplement: Supplementary file 1 [file cells-11-04035-s001.zip › DEGs and enrichment analysis.zip!/DEGs and enrichment analysis/WT_VIMvsVector GO enrichment/DOWN/WT_VIMvsVector_down.dot.pdf]

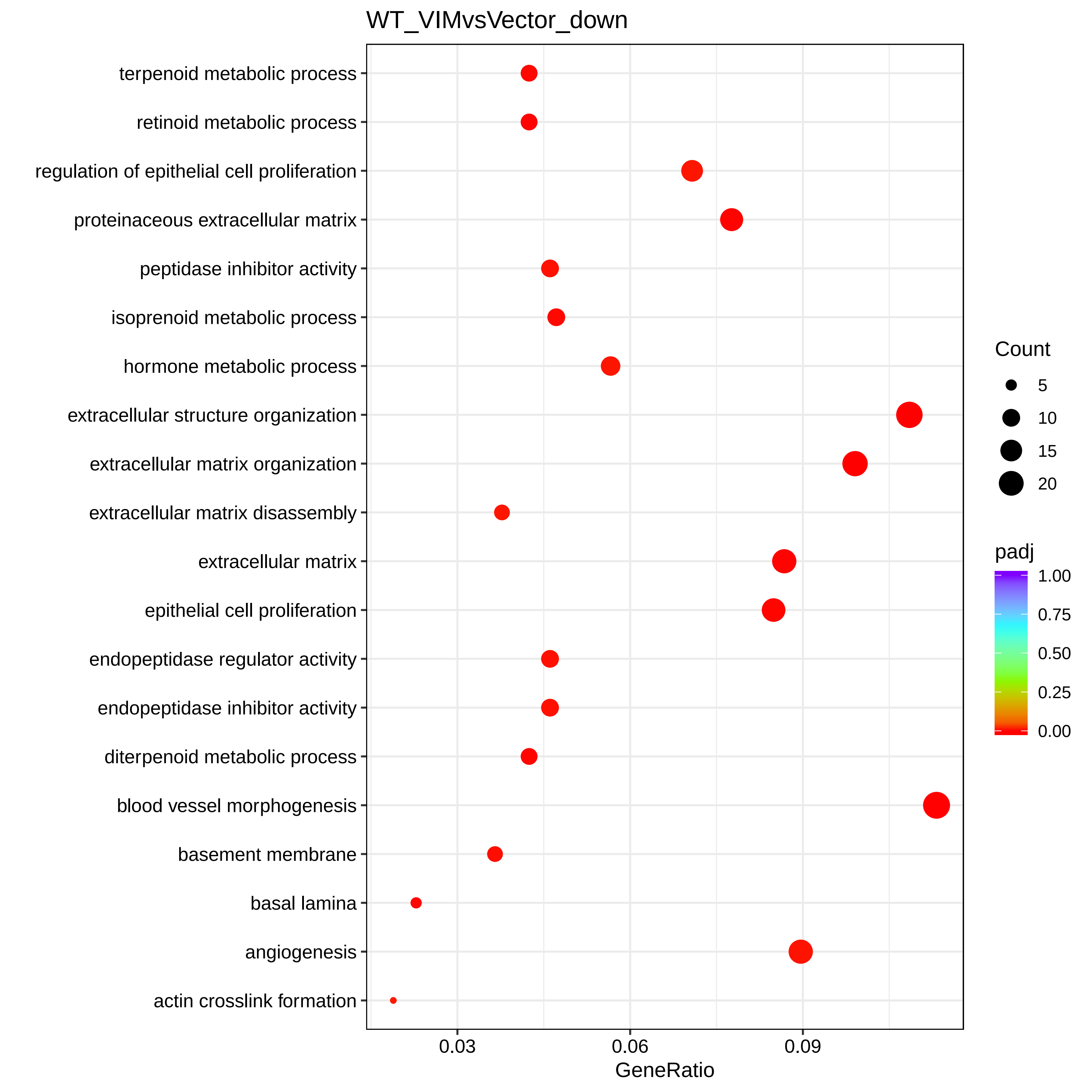
WT_VIMvsVector_down
terpenoid metabolic process
retinoid metabolic process
regulation of epithelial cell proliferation
proteinaceous extracellular matrix
peptidase inhibitor activity
isoprenoid metabolic process
hormone metabolic process
extracellular structure organization
extracellular matrix organization
extracellular matrix disassembly
extracellular matrix
epithelial cell proliferation
endopeptidase regulator activity
endopeptidase inhibitor activity
diterpenoid metabolic process
blood vessel morphogenesis
basement membrane
basal lamina
angiogenesis
actin crosslink formation
0.03
0.06
0.09
GeneRatio
Count
5
10
15
20
padj
1.00
0.75
0.50
0.25
0.00
